# Targeting K-Ras-mediated DNA damage response in radiation oncology: Current status, challenges and future perspectives

**DOI:** 10.1016/j.ctro.2022.10.004

**Published:** 2022-10-17

**Authors:** Mahmoud Toulany

**Affiliations:** Division of Radiobiology & Molecular Environmental Research, Department of Radiation Oncology, University of Tuebingen, Roentgenweg 11, 72076 Tuebingen, Germany

**Keywords:** KRAS, Radiotherapy resistance, Molecular targeting, DNA damage response signaling, Double strand breaks

## Abstract

Approximately 60% of cancer patients receive curative or palliative radiation. Despite the significant role of radiotherapy (RT) as a curative approach for many solid tumors, tumor recurrence occurs, partially because of intrinsic radioresistance. Accumulating evidence indicates that the success of RT is hampered by activation of the DNA damage response (DDR). The intensity of DDR signaling is affected by multiple parameters, *e.g.,* loss-of-function mutations in tumor suppressor genes, gain-of-function mutations in protooncogenes as well as radiation-induced alterations in signal-transduction pathways. Therefore, the response to irradiation differs in tumors of different types, which makes the individualization of RT as a rational but challenging goal. One contributor to tumor cell radiation survival is signaling through the Ras pathway. Three RAS genes encode 4 Ras isoforms: K-Ras4A, K-Ras4B, H-Ras, and N-Ras. RAS family members are found to be mutated in approximately 19% of human cancers. Mutations in RAS lead to constitutive activation of the gene product and activation of multiple Ras-dependent signal-transduction cascades. Preclinical studies have shown that the expression of mutant KRAS affects DDR and increases cell survival after irradiation. Approximately 70% of RAS mutations occur in KRAS. Thus, applying targeted therapies directly against K-Ras as well as K-Ras upstream activators and downstream effectors might be a tumor-specific approach to overcome K-Ras-mediated RT resistance. In this review, the role of K-Ras in the activation of DDR signaling will be summarized. Recent progress in targeting DDR in KRAS-mutated tumors in combination with radiochemotherapy will be discussed.

## 1 Introduction

Conventional fractionated radiotherapy (RT) is a curative approach that significantly contributes to human tumor treatment. Nevertheless, treatment failure still occurs, and overall survival for certain tumor types remains dismal. One of the major mechanisms of tumor survival after irradiation is hyperactivation of the survival signaling pathways regulated by different oncogenes. Among these, RAS (rat sarcoma viral oncogene homolog) encodes a small molecular weight protein with intrinsic GTPase activity. Ras proteins cycle between inactivated GDP-bound and active GTP-bound states and couple extracellular signals to intracellular effector pathways [1]. The three RAS genes in humans consist of KRAS (Kirsten rat sarcoma virus), HRAS (Harvey RAS) and NRAS (Neuroblastoma RAS). The KRAS gene has two splice variants, KRAS4A and KRAS4B, with differential expression levels of the two variants in tumors [2]. Approximately 19 % of human cancers harbor mutations in one of the RAS isoforms, equivalent to approximately 3.4 million new cases per year worldwide [3]. Mutations in RAS genes are mainly at hotspots, such as codons 12, 13 and 61, reviewed elsewhere [4]. Nearly 70 % of the mutations occur in KRAS, mainly in pancreatic cancers (86 %), colorectal cancers (CRC; 41 %) and lung cancer (32 %) [4].

**Fig. 1:**
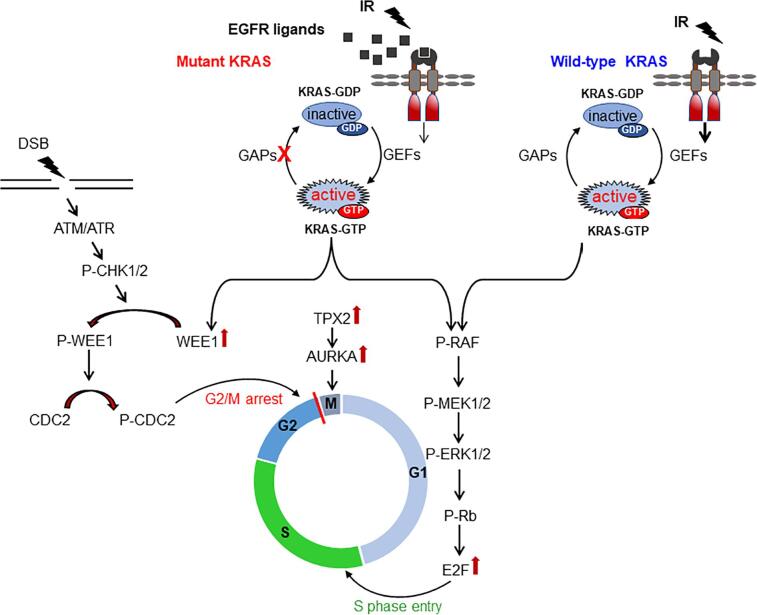
K-Ras controls cell cycle progression after irradiation. A point mutation in KRAS results in autocrine secretion of EGFR ligands but maintains K-Ras in a continuous GTP-bound active state, independent of EGFR. In contrast, ionizing radiation stimulates K-Ras in wild-type cells in an EGFR-dependent manner but independent of the EGFR ligand. Activation of K-Ras stimulates ERK1/2 phosphorylation/activation. ERK1/2 activity stimulates the transition of cells from G1 to S phase. In parallel, KRAS mutation increases the expression of WEE1, which is activated after DNA damage induction. Activated WEE1 phosphorylates CDC2 and prevents mitotic entry by inhibiting the cyclin B/CDC2 complex.

Anchoring Ras to the cell membrane via a variety of posttranslational modifications (PTMs) is essential for the localization of Ras in the cell membrane, subsequent activation and carcinogenic potency. PTMs on K-Ras have been reviewed in [5]. Essential steps in K-Ras PTM are prenylation of CAAX box by the enzymes farnesyltransferase or geranylgeranyl transferase, removing the last 3 amino acids AAX through proteolysis by endoplasmic reticulum located Ras-converting enzyme and, finally, methylation of the cysteine residue of the CAAX box catalyzed by the enzyme isoprenylcysteine carboxyl methyltransferase. Both proteolysis and methylation are essential for efficient membrane binding of prenylated K-Ras [6], a prerequisite for its biological activity. Activation of Ras occurs by changing GDP-bound inactive Ras to the GTP-bound active state. Guanine nucleotide exchange factors (GEFs) and GTPase activating proteins (GAPs), such as neurofibromin 1 (NF1) and p120-GAP, promote nucleotide exchange by Ras (Fig. 1). Due to RAS point mutations, GTPase stimulation by GAPs is greatly reduced, and intrinsic GTPase activity is also reduced. Under these conditions, Ras inactivation is suppressed and it predominantly stays bound to the cell membrane in a constitutively active form [7].

Among the upstream activators of Ras, the epidermal growth factor receptor (EGFR)/HER family consisting of four members, is the most important among the membrane-bound receptor tyrosine kinases. Stimulation of these receptors by their respective ligands results in homo- and heterodimerization, leading to the activation of signaling cascades. Ras transmits these signals, which regulate a variety of cellular functions. Growth, proliferation, survival, tumorigenesis, and metastasis are among the important cellular functions that are stimulated by the EGFR [8]. KRAS mutation leads to constitutive K-Ras activity in association with stimulated autocrine production of the EGFR ligand as reported in panc-1 pancreatic carcinoma cells [9], A549 lung cancer cells and MDA-MB-231 breast cancer cells [10], [11] as well as in CRC cell lines LIM1215, OXCO-2 and DiFi that develop resistance to cetuximab, presumably due to a secondary KRAS mutation[12] (Fig. 1). KRAS mutation promotes post-irradiation cell survival through EGFR activity in non-small-cell lung cancers (NSCLC) as demonstrated in NCI-H1703 cells *in vitro* and *in vivo* [13], [14] and possibly through wild-type HRAS, which has been shown in DLD-1 colorectal and MiaPaCa-2 pancreatic cancer cells harboring frequent mutations in KRAS [15]. Additionally, *in vitro* and *in vivo* studies have shown that EGFR signaling in KRAS-mutant NSCLC cell line A549 promotes chromatin condensation in interphase cells, thereby restricting the number of DNA double-strand breaks (DSB) produced by 1 Gy ionizing radiation (IR) [13]. In addition to point mutations and stimulation with receptor ligands, exposure to a clinically relevant dose of IR rapidly activates K-Ras in KRAS wild-type head and neck squamous cell carcinoma FaDu cells [10].

GTP-bound Ras stimulates several cytoplasmic signaling cascades. Among these pathways, mitogen-activated protein kinase (MAPK), phosphoinositide 3-kinase (PI3K), protein kinase C and RAL guanine nucleotide dissociation stimulator (RALGDS) are the most important pathways [16], [17]. K-Ras triggers the Raf family of serine/threonine kinases, which in turn stimulates mitogen-activated protein kinase kinase 1/2 (MEK1/2). MEK1/2 stimulate MAPK/extracellular signal-regulated kinases (ERK1/2). Activated ERK1/2 phosphorylate their target substrates, such as multifunctional protein Y-Box binding protein-1 (YB-1) [18], [19]. Oncogenic K-Ras hyperactivates the PI3K/AKT pathway. The PI3K/AKT pathway is the major survival pathway, which is hyperactivated in human tumors and is involved in DNA damage response (DDR) signaling, as reviewed elsewhere [20], [21]. In terms of cell survival after RT, constitutive K-Ras activity due to RAS mutation or IR-induced Ras activation leads to accelerated repair of radiation-induced DSB and increased survival in solid tumors from different tissues [18], [22], [23], [24], [25]. In line with the preclinical data on the role of K-Ras in therapy resistance, the association of KRAS mutation with worse treatment outcome has been well documented. In this regard, pancreatic cancers, as the most lethal cancer with frequent mutations in KRAS, have a 5-year survival rate of approximately 10 % [26]. In a study in Chinese patients with advanced pancreatic ductal adenocarcinoma, patients with KRAS mutation showed worse overall survival than patients with KRAS wild-type [27]. Likewise, KRAS wild type pancreatic adenocarcinoma patients exibited a survival benefit, both, in overall cohorts and in patients treated with chemotherapy agents [28]. The second most common KRAS-mutated human tumor is CRC, which is resistant to chemoradiation [29], [30]. KRAS-mutated cervical cancer has significantly worse recurrence-free survival and distant metastases after RT [31]. The lung adenocarcinoma subgroup of NSCLC is the third cancer category with the most frequent mutations in KRAS. NSCLC has a 5-year survival rate of approximately 25 %, and KRAS mutation has been shown to be associated with radioresistance [32] and decreased cancer-specific survival after lung stereotactic RT [33]. Thus, accumulating evidence from preclinical and clinical studies indicates that mutation in KRAS diminishes the effect of RT.

## 2 Role of K-Ras in DDR signaling

### 2.1 K-Ras cascades regulate cell-cycle progression

Upon induction of DNA damage, the DDR is activated, which is a complex signal-transduction network responsible for sensing and responding to specific types of DNA damage, encompassing specific machineries mediating cell-cycle regulation and DNA repair. Exposure to IR (2 Gy) rapidly stimulates Ras-dependent activation of ERK1/2 by phosphorylation of the threonine and tyrosine residues in different tumor cells lacking mutations in the components of the Ras/MAPK pathway [34], [35]. ERK1/2 activity stimulates the expression of immediate target genes, which in turn, by phosphorylating retinoblastoma protein (RB), enhances the expression of the transcription factor E2F and prepares cells for transition from the G1 to S phase [36] along with sequestering and degrading cyclin-dependent kinases (CDKs), such as p27 [37], [38]. ERK1/2, as a regulator of the G1- to S-phase transition, has been extensively reviewed previously [39]. Continuous activation of ERK1/2, in parallel to stimulating the expression of proliferation-associated genes, suppresses the expression of antiproliferative genes as well [40], [41]. IR-induced ERK phosphorylation promotes cell proliferation by stimulating the G1 to S transition, as shown in KRAS^G13D^-mutated colorectal cancer HCT116 cells after irradiation with 1 Gy [17]. The transition of cells from G1 to S after irradiation is important in the context of DSB repair. Radiation-induced DSB in G1 phase in cells with wild-type TP53 results in ataxia telangiectasia mutated (ATM)/ checkpoint kinase 2 (CHK2)-dependent G1 arrest until the damage is repaired through NHEJ. G1 progression in cells with loss of p53 function makes DSB repair solely dependent on G2/M arrest. G2/M arrest through ataxia telangiectasia and Rad3 related (ATR)/checkpoint kinase 1 (CHK1) arrest is crucial since the majority of tumors with KRAS mutations also harbor TP53 mutations, as shown in pancreatic ductal carcinomas [42]. The expression of mutated KRAS and TP53 in NSCLC was also shown to be associated with poor clinical outcomes [43]. Due to the role of CHK1 in G2 arrest and, consequently, HR repair of DSB, CHK1 and ATR have been described to be targets in combination with DNA damage-inducing agents as treatment approaches for pancreatic cancers [44], [45]. The p53-independent expression of the CDK inhibitor p21 in response to mitogen-activated ERK1/2 has been described in early G1 phase. However, it remains to be investigated whether in TP53-mutated cells IR-induced activation of ERK1/2 also induces p21 expression [40]. Hyperactivation of ERK1/2 in KRAS-mutated cells also stimulates cell proliferation. In this context, the stimulated proliferation of KRAS^G12D^-transformed pancreatic ductal cells harboring common TP53 and p16(INK4a) mutations was shown to be dependent on ERK2 downstream of K-Ras [46].

KRAS mutation increases the expression of WEE1 kinase, which drives cell-cycle progression [47]. Upon induction of DNA damage, WEE1 becomes activated, presumably by CHK1 and CHK2 as ATR and ATM downstream kinases [48]. Mitotic entry is prevented by inhibition of the cyclin-B/CDC2 complex, in which inhibitory phosphorylation of CDC2 plays a fundamental role and is stimulated by WEE1 activity [49]. Thus, the function of WEE1 in G2/M arrest is crucial in all tumor cells, especially in TP53-mutated tumors with a lack of G1 arrest. Accordingly, targeting WEE1 can be an efficient approach to sensitize tumor cells to DNA-damaging agents [50], [51], particularly in TP53-mutated cells, based on the concept of synthetic lethality [52].

Aurora kinase A (AURKA) is involved in the G2/M transition by promoting centrosome maturation and mitotic spindle assembly and is thus involved in chromosomal stability [53]. Oncogenic K-Ras stimulates the expression of both AURKA and its activator,TPX2, as shown in pancreatic ductal adenocarcinomas (PDACs) cell lines KRAS^G12D^ mutated PANC-1 and KRAS^G12C^ mutated PaCa-2 [54]. In PDACs, high expression levels of AURKA and TPX2 were associated with shorter patient survival and the presence of oncogenic KRAS [54]. The expression of AURKA is stimulated by IR in a dose-dependent manner and induces radioresistance in preclinical studies [55], [56], [57]. Likewise, it has been shown that AURKA expression diminishes RT outcome in cancer patients with different tumor types [58], [59], [60]. Thus, it can be concluded that K-Ras-mediated radiochemotherapy resistance occurs partially through regulating cell-cycle progression. The function of oncogenic K-Ras in cell-cycle progression is outlined in Fig. 1.

### 2.2 K-Ras downstream cascades stimulate DNA DSB repair

**Fig. 2:**
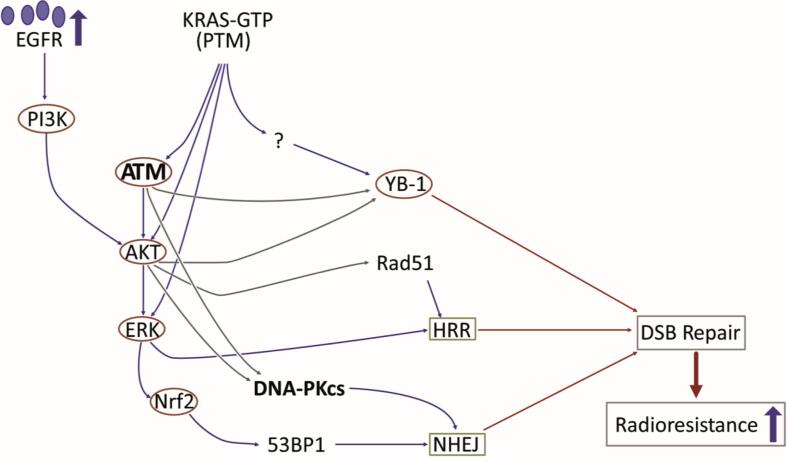
K-Ras stimulates repair of IR-induced DSB. The PI3K/AKT and MAPK/ERK pathways are the major signaling pathways activated either directly by K-Ras-GTP or downstream to EGFR by K-Ras-dependent autocrine secretion of EGFR ligands. Activation of these two pathways accelerates DSB repair by stimulating key components of the HR and NHEJ repair pathways. For further details, please see the text.

Direct evidence for oncogenic K-Ras-mediating radioresistance was initially shown by Bernhard et al. [24] and Kim et al. [61], who demonstrated that knockdown of K-Ras by siRNA in KRAS^G12V^ SW480 cells resulted in radiosensitization by a radiation dose enhancement ratio (DER) of 1.22. PI3K/AKT and MAPK/ERK are the two major pathways downstream of K-Ras that are hyperactivated by mutated KRAS and stimulated by irradiation. The impact of these two pathways on DDR repair has been investigated mainly in the context of stimulating the 3 key kinases, *i.e.*, ATM, DNA-dependent protein kinase catalytic subunit (DNA-PKcs) and ATR. Activation of ATM following 53BP1 focus formation at the DNA damage site seems to be one of the early steps in the DDR after irradiation [62]. ATM induces CHK2 activity, leading to p53-dependent responses that promote G1 cell-cycle arrest, as a prerequisite event for DSB repair through NHEJ. Activation of p53 also induces chromatin remodeling and enhances the induction of DNA-repair genes [63]. If radiation-induced DNA damage is not repaired, cell death occurs mainly due to mitotic catastrophe but not senescence or autophagy [64], [65]. ATM functionally interacts with K-Ras. ATM deficiency markedly increases the proportion of chromosomal alterations in pancreatic primary tumors with KRAS^G12D^ and renders pancreatic tumors highly sensitive to radiation in association with increased residual DSB 24 h post-4 Gy [66]. This observation indicates a link between the K-Ras protooncogene and the activation of ATM. Several studies have shown that the activation of ERK and AKT depends on ATM kinase activity and that ATM forms a complex with ERK1/2 [67]. Thus, the K-Ras-ATM-AKT-ERK signaling pathway can be one of the underlying signaling pathways involved in DSB repair in KRAS-mutated cells. This conclusion is supported by the literature indicating that DSB repair is stimulated by HR and NHEJ through the AKT and ERK pathways [68], [69], [70], [71], [72], [73], [74], [75]. In further support of this conclusion, ATM was shown to mediate crosstalk between the prosurvival MEK/ERK and AKT/mTOR pathways as the two major pathways regulated by K-Ras [76]. The key function of K-Ras in this interaction is also supported by the enhanced sensitivity of KRAS-mutant lung cancer cells to MEK inhibition after ATM loss [76] and radiosensitization of KRAS-mutated pancreatic cancer cells after MEK targeting through inhibition of HR- and NHEJ-dependent DSB repair [77]. KRAS mutation also activates NRF2 antioxidant signaling [78], [79] that suppresses p53 expression [78] and increases the expression of 53BP1 [79]. Upregulation of 53BP1 stimulates NHEJ repair and mediates radioresistance, as shown in CRC cells SW48 and HCT116 after single doses of IR up to 8 Gy [79]. In KRAS^G12V^-mutated NSCLC cell line A549 cells, AKT1-dependent expression of RAD51 has also been reported [80]. This indicates that KRAS-mutated cells may produce an effective HR repair of DSB, as described previously in lung cancer cells [81] (Fig. 2).

YB-1 is highly expressed in human solid tumors from different tissues and is involved in various cellular processes, *e.g.*, cell cycle progression, maintaining cancer stemness and DDR as reviewed in [82], [83]. The cancer hallmarks described thus far, *i.e.*, resisting cell death such as after RT, are also regulated by YB-1 [84]. YB-1 expression is associated with a limited response to radiochemotherapy [18], [83], [85]. Serine 102 (S102) is one of the major phosphorylation sites in YB-1 that is necessary for its cellular functions. p90 ribosomal S6 kinase (RSK) and AKT are the key kinases within the oncogenic K-Ras downstream pathway that directly activate YB-1 [18], [86]. YB-1 is constitutively phosphorylated at S102 in KRAS-mutated tumor cells, *i.e.*, in CRC cells [85] and in triple-negative breast cancer (TNBC) cells [18]. In KRAS wild-type breast cancer cells, exposure to IR (4 Gy) induces YB-1 phosphorylation as early as 5 min post-IR [18]. Delayed YB-1 phosphorylation was also detected in CRC cells 6 h as well as 12 h after IR (5 Gy) [87]. Similar to the knockdown of K-Ras, knockdown of YB-1 inhibits the repair of IR-induced DSB in KRAS^G13D^-mutated MDA-MB-231 breast cancer cells after irradiation with single doses of 2, 4 and 6 Gy, resulting in an enhanced radiosensitivity [18]. Likewise, blocking YB-1 with an S102 blocking peptide [83] or by impairing its interaction with RSK [88] blocks DSB repair after 4 Gy irradiation in breast cancer cells. Thus, YB-1 is one of the components that is stimulated by the K-Ras protooncogene and enhances DSB repair, mediating RT resistance. The function of oncogenic K-Ras in DSB repair is outlined in Fig. 2.

## 3 Targeting strategies to improve RT of KRAS-mutated tumors

### 3.1 Targeting the K-Ras-regulated components of DDR signaling

As reviewed above, KRAS mutation interferes with cell-cycle progression by stimulating the G1 transition and activating G2/M arrest (Fig. 1). After induction of DSB, arrested cells initiate DSB repair through HR and NHEJ in KRAS-mutated cells (Fig. 2). According to the function of K-Ras in DDR signaling, targeting K-Ras signaling will partially interfere with DSB repair after irradiation either by overwhelming cell-cycle arrest or by hampering the components of repair pathways downstream of K-Ras. Thus, a selective approach to overcome RT resistance of KRAS-mutated cells would be targeting the components of cell-cycle progression and DNA repair that are known to be affected by mutated KRAS. In preclinical studies, the classical components of cell-cycle progression and DNA-damage repair, such as ATM, DNA-PKcs, ATR, CHK1, CHK2 and WEE1, have shown to be very promising targets for radiosensitizing tumor cells by applying small-molecule inhibitors [89], [90], [91], [92]. Since the majority of tumors with KRAS mutations also harbor *TP53* mutations, targeting the G2/M checkpoint might be the most effective approach to interfere with the repair of DSBs in KRAS-mutated cells. Thus, among a variety of targets involved in DDR and regulated by K-Ras, the current status of targeting DDR signaling involved in the G2/M checkpoint will be summarized. The major focus will be on those clinical trials performed specifically in 3 tumor entities, *i.e.,* pancreatic, CRC and lung cancers with frequent mutations in the KRAS gene.

#### 3.1.1 Targeting ATR

ATR is activated upon DNA damage and in turn activates CHK1 to induce G2/M cell-cycle arrest. The mechanism of ATR function in DDR has been extensively investigated in preclinical studies. Currently, specific small-molecule inhibitors of ATR, *e.g.*, VE-821, M6620 (berzosertib, VX-970), and AZD6738 (ceralasertib), are available, and the sensitizing effect of ATR inhibitors has been successfully demonstrated after combination with RT or chemotherapy agents. In PSN-1, MiaPaCa-2 and primary PancM pancreatic cancer cells, VE-821, as the first highly selective and potent ATR inhibitor, increased sensitivity to radiation doses of 2, 4 and 6 Gy *in vitro* [93]*.* VE-821 also induced sensitivity of the pancreatic cancer cells to gemcitabine [93]. Likewise, ATR inhibition by VE-821 enhanced the response to a single dose irradiation of 6 Gy in a PSN-1 tumor xenograft model *in vivo* [94]. A study by Baschnagel *et al.* demonstrated that inhibition of ATR by M6620 radiosensitizes NSCLC brain metastasis patient-derived xenografts [95].

#### 3.1.2 Targeting CHK1

ATR stimulating CHK1 activation protects oncogenic K-Ras-expressing pancreatic cancer cells from DNA damage induced by the irradiation mimic neocarzinostatin [44], potentially through interference with G2 arrest and successful DSB repair. AZD7762, MK-8776 (SCH 900776) and LY2606368 (prexasertib) are the most studied CHK1 inhibitors that have been investigated in combination with DNA-damaging agents in a variety of tumor entities. In a study by Dinkelborg et al, targeting CHK1 in combination with single doses of irradiation (2, 4, 6, and 8 Gy) resulted in radiosensitization in KRAS mutant/hyperactivated TNBC cells but not in KRAS wild-type non-TNBC cells. [96]. Targeting CHK1 as a potential approach to induce radiosensitization of KRAS-mutated cells has also been demonstrated in tumor cells with frequent KRAS mutations. In pancreatic cancer cells, targeting CHK1 by applying AZD7762 or MK-8776 was shown to improve the effect of chemotherapy agents [97], [98], radiation [99], [100], and chemoradiation [101] *in vitro* and/or *in vivo*. In KRAS-mutated rectal cancer cells, AZD7762 was shown to be an effective compound to induce radiosensitization after 2 Gy irradiation [102]. Likewise, lung cancer cells could also be radiosensitized by CHK1 inhibitors in preclinical studies *in vitro* and *in vivo* [103], [104]. Currently, there are no clinical studies published applying CHK1 inhibitors in combination with chemoradiation in tumors with frequent KRAS mutations. However, based on the results published from preclinical studies, the combination of CHK1 inhibitors in patients with KRAS-mutated tumors may be beneficial and should be tested in a clinical setting. Table 1 presents the clinical trials in which CHK1 targeting was combined with chemotherapy or RT in 3 tumor entities most frequently harboring KRAS mutations, *i.e.*, pancreatic cancer, CRC and lung cancers.

**Table 1:** Clinical studies targeting K-Ras and K-Ras-regulated DDR signaling. The studies in combination with RT are marked in bold.

**Target**	**Drug**	**Trial**	**Combination**	**Cancer type**	**Major findings**	**Ref.**
ATR	M6620	phase I	*GEM*	advanced NSCLC	- well tolerated.	[129]
			+/- CB	ST including CRC	- well tolerated. - complete response in ATM loss CRC	[130]
			TPT	ST including NSCLC and PaCa	- MTD of combination was well tolerated - enhanced DNA DSB in combination - partially active in TPT-non-responding NSCLC	[131]
CHK1	AZD7762	phase I	*GEM*	ST including CRC and lung cancer	- MTD of 21 mg, stable disease	[132]
		phase II	*GEM*	PaCa	- not superior to *GEM*	[133]
	LY26063618	phase II	*GEM* + CDDP	advanced nonsquamous NSCLC	- improved PFS - increased risk of thromboembolism	[134]
			pemetrexed	advanced or metastatic NSCLC	- partial response (9.1 %) - stable disease (36.4 %) - no association between p53 status and response	[135]
WEE1	AZD1775	phase II	***GEM* + RT**	**LAPaCa**	- **well tolerated** - **improved OS**	[110]
			–	RAS and TP53 mutations mCRC	- improved PFS	[109]
FTase	tipifarnib	phase I	–	brainstem glioma	- MTD (125 mg/m^2^ twice-daily)	[136]
			**RT+/-TMZ**	**GBM**	- **MTD (300 mg/m^2^ twice-daily)**	[137]
			**RT**	**GBM**	- **MTD (200 mg/m^2^/day)**	[138]
		phase II	**RT**	**brainstem glioma**	- **no clinical advantage**	[115]
				**GBM**	- **no clinical advantage**	[116]
FTase + GGTase-1	L-778,123	phase I	**RT**	**HNSCC and NSCLC**	- **acceptable toxicity** - **partial to complete response** - **radiosensitization of PD cell line** - **accumulated in G2/M after L-778,123**	[118]
				**PaCa**	- **acceptable toxicity.** - **radiosensitization of PD cell line**	[119]
KRAS^G12C^	AMG 510	phase I	–	KRAS ^G12C^ ST	- encouraging anticancer activity - Grade 3 or 4 treatment-related toxic effects occurred in 11.6 %	[128]
		phase II	–	KRAS ^G12C^ NSCLC, previously treated with standard therapies	- durable clinical benefit - partial and complete response in 37.1 % - adverse events in 69.8 %	[139]
				KRAS ^G12C^ CRC, previously treated with standard therapies	- 9.7 % overall response	[140]

**ATR:** ataxia telangiectasia and Rad3 related, **CB**: carboplatin, **CDDP:***cis*-diammindichloridoplatin, **CHK1:** checkpoint kinase 1, **CRC:** colorectal cancer, **DSB:** double-strand break, **FTase:** farnesyltransferase**, GBM:** glioblastoma multiforme, ***GEM*:** gemcitabine, **GGTase-1:** geranylgeranyl transferase type-1, **HNSCC:** head and neck squamous cell carcinoma**, LAPaCa:** locally advanced pancreatic cancer, **mCRC:** metastatic colorectal cancer **MTD:** maximum tolerated dose, **NSCLC:** non-small-cell lung cancer, **PaCa:** pancreatic cancer, **PD:** patient derived, **PFS:** progression-free survival, **OS:** overall survival, **RT:** radiotherapy, **ST:** solid tumors, **TMZ:** temozolomide, **TPT:** topotecan.

#### 3.1.3 Targeting WEE1

WEE1 is the key kinase and the direct target of CHK1, and its expression is enhanced by KRAS mutation, as shown in pancreatic cancer cells [47]. In irradiated cells, activation of WEE1 stimulates G2/M cell-cycle arrest and, consequently, DSB repair through both HR and NHEJ. Thus, WEE1 inhibition can boost the cells harboring residual DSB through replication, resulting in cell death through mitotic catastrophe. The WEE1 kinase inhibitor AZD1775 (adavosertib) has been investigated in preclinical studies and has shown radio/chemosensitization. In pancreatic cancer cells, as a representative KRAS-mutated tumor entity, targeting WEE1 induced sensitization to radiation with a DER of 1.3 ± 0.1 in MiaPaCa-2 cells and gemcitabine chemoradiation *in vitro* and *in vivo* through inhibition of the HR repair pathway and abrogation of the G2 checkpoint [105], [106]. Abrogation of the G2 checkpoint as a potential mechanism of radiosensitization in pancreatic cancer cells has also been shown *in vitro* and *in vivo* through controlling translational regulation of WEE1 and RAD51 by metformin [107]. Furthermore, WEE1 targeting was shown to be more efficient in KRAS-mutant NSCLC expressing mutated TP53 [108], indicating that the G2 checkpoint is the major target of WEE1 inhibitors. The data obtained from clinical trials of the WEE1 kinase inhibitor AZD1775 are promising. In a phase II randomized trial, AZD1775 improved the progression-free survival of CRC patients with KRAS- and TP53-mutated tumors and was well-tolerated [109]. Together, pharmacological inhibitors targeting the components of the ATR/CHK1/WEE1 pathway in 3 tumor entities expressing most KRAS mutations have reached phase II clinical trials. However, except for one study, nearly all other studies were in combination with chemotherapy, and combination studies with RT or radiochemotherapy should be conducted. To date, the data of only one study have been published combining the WEE1 inhibitor AZD1775 with gemcitabine and RT in pancreatic cancers [110]. In this study, AZD1775 in combination with gemcitabine and RT was well tolerated, and the overall survival was described to be substantially higher than prior results combining gemcitabine with radiation [110]. The result of this study [110] is of special importance because it targets WEE1, whose expression is expected to be higher in KRAS-mutated tumors than in KRAS wild-type tumors [47]. This difference might be an advantage for targeting WEE1 instead of ATM and CHK2 to block G2/M arrest in terms of tumor specificity and limited normal tissue toxicity issues.

### 3.2 Direct targeting of KRAS PTMs

Prenylation, palmitoylation/depalmitoylation, phosphorylation, acetylation, nitrosylation, ubiquitination and SUMOylation are the major PTMs of K-Ras [5]. These PTMs regulate K-Ras membrane localization and, consequently, its activity and oncogenic capacity. Thus, inhibitors of PTMs were supposed to be the first approach to overcome the radioresistance of KRAS-mutated tumors. Prenylation by farnesyl-protein transferase as the first step in K-Ras maturation occurs by adding the lipid farnesyl group to the cysteine near the end of the target protein, which is necessary for localization of K-Ras to the plasma membrane [111]. Targeting farnesyltransferase was one of the first approaches proposed to target Ras by applying farnesyltransferase inhibitors (FTIs). Several FTIs, *i.e.*, L-744,832, L-778,123, FTI-276 and R115777 (tipifarnib), have been preclinically investigated in combination with RT. A study by Cohen-Jonathan et al. was one of the first studies to demonstrate that inhibition of farnesyltransferase by L-744,832 and FTI-276 radiosensitizes T24 bladder cancer cell lines expressing HRAS mutations but not the human colon carcinoma cell line HT-29, which expresses wild-type RAS, and both cell lines express TP53 mutations [112]. A separate study from the same group demonstrated that FTI can change the oxygenation of HRAS mutated but not HRAS wild-type tumors [113]. In a further study of L-744,832 in pancreatic cancer cells, it was demonstrated that L-744,832 enhanced the cytotoxic effect of IR, apparently by overriding G2/M checkpoint activation [114]. Although the effect of the studied FTIs on DSB repair has not been investigated in any of these studies, the results confirmed the potential radiosensitizing effect of FTIs in pancreatic cancer in combination with clinically relevant doses of IR. However, cell-cycle regulation by L-744,832 was associated with changes in PTM of H-Ras and *N*-Ras, but not K-Ras [114]. From this study, it could be concluded that FTIs can also interfere with the activation and function of other farnesylated proteins.

Most clinical trials on the combination of FTIs with RT have been conducted in brain tumors (Table 1). Tipifarnib entered a phase II trial in combination with RT of gliomas and glioblastomas [115], [116]. Administration of RT did not offer a clinical advantage over historical controls in pontine gliomas [115]. A similar result was observed from the combination of tipifarnib with RT in glioblastomas. The lack of benefit of the combination of tipifarnib with RT in brain tumors is not surprising since only 2 % of glioblastomas harbor RAS mutations [117]. Thus, RAS mutation seems to be a prerequisite for selecting patients for the combination of RT and FTIs. This conclusion is supported by the results of the combination of FTI L-778,123 in NSCLC [118] and pancreatic cancers [119]. Although RAS mutation was not essential for study enrollment, L-778,123 in a phase I trial led to local responses without an increase in RT-associated toxicities in four NSCLC patients [118]. In a study of four pancreatic cancers (3 with KRAS mutation), a combination of L-778,123 and RT at dose level 1 showed acceptable toxicity. In this study, L-778,123 radiosensitized a patient-derived cancer cell line [119].

Since only very limited clinical trials on the combination of FTIs with RT exist, no solid conclusion can be made on the effectiveness of such a combination. Thus, the combination of FTIs with standard RT is reasonable, and in such a trial, stratification on the basis of mutation or pathway activation would be more informative. Evidence from preclinical studies indicates that in farnesyltransferase-inhibited cells, prenylation of K-Ras can occur by geranylgeranyl transferase type-1 (GGTase-1) [120]. Therefore, the applied FTI should also be able to block GGTase-1 with tolerable toxicity when combined with RT.

### 3.3 Other approaches to block prosurvival effect of K-Ras protooncogene

Discovering small-molecule inhibitors that bind irreversibly in the switch-II pocket of oncogenic KRAS^G12C^ [121], [122] accounts for the latest progress in targeting the K-Ras protooncogene. AMG510 (sotorasib) is among those K-Ras^G12C^ inhibitors that has been approved by the FDA for the treatment of patients with NSCLC with KRAS^G12C^ mutations. To date, most of the studies with AMG510 have been performed on the antitumor activity of the compound as a single treatment or in combination with DNA damage-inducing chemotherapy agents. The mutation-specific cellular activity of AMG510 has been shown across a panel of KRAS^G12C^-mutated cell lines compared to non-KRAS^G12C^ lines [123]. AMG510 was shown to selectively target KRAS^G12C^ mutated tumors, with a durable tumor regression effect as a monotherapy and with a synergistic effect in combination with chemotherapy and targeted therapy agents [123]. Several clinical trials with AMG510 are enrolling patients for study. The results of previously published trials are summarized in Table 1. The effect of K-Ras^G12C^ inhibitors in terms of an objective response in NSCLC and CRC patients is limited, which may be due to the reported resistance to these inhibitors. In this context, the development of resistance to AMG510 has been demonstrated to be due to a variety of different mechanisms. Deregulation of upstream receptor tyrosine kinases, activation of the MAPK/ERK and PI3K/AKT pathways due to mutations in the regulatory components of these pathways, KRAS secondary mutation in codons 12, 13, 61, 68, 95, 96 and amplification of the KRAS^G12C^ allele are among those resistance mechanisms [124], [125], [126], [127]. Additionally, applying these inhibitors is limited to a small number of tumors with KRAS mutation, *i.e.*, in 13 % of KRAS mutated NSCLCs, in 1 to 3 % of KRAS mutated colorectal cancers and in 1 to 2 % of KRAS mutated pancreatic cancers [128]. Thus, due to the diverse mechanisms of resistance to KRAS^G12C^ inhibitors and applicability of this strategy to very limited tumors, there is uncertainty regarding the combination of this strategy with radiotherapy.

Targeting upstream and downstream effectors of K-Ras, *e.g.*, EGFR, PI3K, Raf, AKT and MEK, is not restricted to KRAS-mutated cells. These targeting strategies were not discussed in this review since they have been extensively reviewed by other investigators.

## 4 Conclusions and future directions

In addition to targeting the components of K-Ras downstream cascades, two major strategies have been used to overcome K-Ras-induced radioresistance, which were reviewed here. Targeting the PTM of K-Ras is the most well-studied approach. In this context, preclinical studies showed that the use of FTI in combination with RT may be an effective approach to radiosensitize KRAS-mutated cells. However, the applied FTIs should also be able to block prenylation of K-Ras not only by blocking farnesyltransferase but also by inhibiting GGTase-1 as an acquired resistance mechanism to FTIs. Due to the lack of benefit of the combination of FTIs with RT of KRAS wild-type tumors, KRAS mutation should be a prerequisite for enrolling patients in future trials.

The role of the protooncogene K-Ras in the DNA-damage response and G2/M cell-cycle arrest has been well described. This function of KRAS may well be of special importance in tumors with TP53 mutations that rely on the G2/M checkpoint for DNA repair after RT, based on the concept of synthetic lethality. The results of early-phase clinical trials applying inhibitors of ATR, CHK1 and WEE1 are promising and suggest that suppressing G2/M arrest might be the most effective approach to combine with RT in KRAS-mutated tumors.

## Financial support

The authoŕs research is supported by the German Research Foundation (Deutsche Forschungsgemeinschaft, DFG TO 685/2-3 and TO 685/6-1).

## Declaration of Competing Interest

The authors declare that they have no known competing financial interests or personal relationships that could have appeared to influence the work reported in this paper.
